# Methicillin-resistant *Staphylococcus aureus* from infected skin lesions present several virulence genes and are associated with the CC30 in Brazilian children with atopic dermatitis

**DOI:** 10.1080/21505594.2020.1869484

**Published:** 2021-01-07

**Authors:** Fernanda Sampaio Cavalcante, Simone Saintive, Dennis Carvalho Ferreira, Adriana Barbosa Rocha Silva, Lorrayne Cardoso Guimarães, Beatriz Stofel Braga, Eliane de Dios Abad, Marcia Ribeiro, Kátia Regina Netto dos Santos

**Affiliations:** a Campus Macaé, Universidade Federal do Rio de Janeiro, Macaé, Brazil; b Instituto de Puericultura e Pediatria Martagão Gesteira, Universidade Federal do Rio de Janeiro, Rio de Janeiro, Brazil; c Faculdade de Odontologia, Universidade Veiga de Almeida, Rio de Janeiro, Brazil; d Faculdade de Odontologia, Universidade Estácio de Sá, Rio de Janeiro, Brazil; e Instituto de Microbiologia Prof. Paulo de Góes, Universidade Federal do Rio de Janeiro, Rio de Janeiro, Brazil

**Keywords:** Atopic dermatitis, *S. aureus*, virulence, methicillin resistance, clonal complex 30

## Abstract

Atopic dermatitis (AD) is a chronic inflammatory skin disease and colonization by *Staphylococcus aureus* may affect up to 100% of these patients. Virulent and resistant isolates can worsen AD patient clinical condition and jeopardize the treatment. We aimed to detect virulence genes and to evaluate the biofilm production of *S. aureus* isolates from infected skin lesions of children with AD. Methicillin resistance was detected by phenotypic and molecular tests and the virulence genes were detected by PCR. Biofilm formation was assessed by bacterial growing on microtiter plates and later stained with safranin. Genotyping was performed by Pulsed-Field Gel Electrophoresis and Multilocus Sequence Typing. Among 106 AD patients, 55 (51.8%) had developed *S. aureus* cutaneous infections and 23 (41.6%) were methicillin-resistant (MRSA). All 55 isolates carried the *fnbA, hla, icaA, sasG,* and *seu* genes, and more than 70% presented *cna, eap, ebpS, hlg,* and *pvl* genes. Clonal complex (CC) 30 was the main lineage found (34.5%), especially among MRSA isolates (52.2%). The *egc* cluster and the *bbp* gene were significantly the most frequent in MRSA isolates and in USA1100/ST30/CC30 lineage. Most of the isolates (74.5%) were non-biofilm producers and many of them only started to produce it in the presence of fibrinogen. There was no significant association between *S. aureus* isolates features and the AD severity. This study demonstrated a high frequency of CC30 MRSA isolates presenting several virulence genes in infected skin lesions of AD children in Brazil, that may influence the severity of the disease and the treatments required.

## Introduction

Atopic dermatitis (AD) is a chronic skin disorder which the main signs and symptoms are pruritus, xerosis, and eczematous lesions placed on hands, neck, popliteal, and antecubital fossae [1]. The severity of AD can be classified by the SCORAD (scoring atopic dermatitis) index, which is based on lesions and symptoms extension and intensity, and can be classified as mild (SCORAD <25), moderate (25–50) or severe (>50) [2]. Genetic predisposition, skin barrier disruption, exposure to environmental factors, and dysbiosis seem to be associated with the disease [3]. Skin colonization by *S. aureus* may contribute to the onset and/or aggravation of these lesions [4]. Prevalence of this pathogen in AD skin lesions may affect up to 100% of these patients [5,6] and consequently, *S. aureus* infections are the most common aggravating factor of AD. Nevertheless, molecular epidemiology of *S. aureus* from infected AD lesions, especially in developing countries, remains unknown.

Exacerbations of AD caused by *S. aureus* are probably due to staphylococcal toxins that can aggravate the eczema [7]. Furthermore, staphylococcal superantigens (SAgs) have been shown to rapidly induce the IL31 mRNA expression in the skin of atopic subjects *in vivo* and in peripheral blood mononuclear cells *in vitro*, suggesting that chronic colonization and super infection by *S. aureus* can contribute to pruritus and inflammatory changes in patients with AD [8].

*S. aureus* presents a great number of virulence factors, including secreted proteins and enzymes that might be used to establish and maintain the infection [9]. Some studies have evaluated isolates recovered from skin of AD patients and found genes of SAgs such as the toxic shock syndrome toxin-1 (TSST-1), staphylococcal enterotoxins (SEs), and the SE-like proteins [10–13]. Other exotoxins described in *S. aureus* isolates from AD patients include: cytolysins, particularly the α-, β-, γ-, and δ-toxins; phenol soluble modulins and leukocidins [9]. Also, some *S. aureus* isolates from AD skin lesions have been shown to produce biofilm, which could be associated with inflammation and pruritus probably due to the occlusion of sweat ducts [14].

As previous explained, *S. aureus* colonization can aggravate AD and the virulence factors produced by this pathogen can play a significant role in disease manifestations. Therefore, the aim of this study was to assess the characteristics associated with virulence, antimicrobial resistance, and clonality of the *S. aureus* isolates from infected skin lesions of Brazilian children with AD, and to clarify the relationship of these characteristics with the severity of the disease.

## Materials and methods

### Clinical isolates, setting and ethics statement

A cross-sectional study was conducted, between September 2011 and September 2013, at the pediatric dermatology outpatient clinic of the Instituto de Puericultura e Pediatria Martagão Gesteira (IPPMG) of the Universidade Federal do Rio de Janeiro (UFRJ), Brazil, where 130 AD pediatric patients were being accompanied at the time attended. The target population of the study included male or female patients diagnosed with AD who were 15 years old or less. The study was approved by the ethical committee of the IPPMG (Nº 51/11).

Swabs of skin infected lesions were obtained from all patients clinically diagnosed by pediatric dermatologists [15]. In brief, a sterile swab moistened in saline solution (0,85%) was placed on the skin injury, rotated three times, and plated onto mannitol salt agar (Oxoid; Basingstoke, United Kingdom). The plates were incubated for 48 h at 35°C and the bacterial isolates were characterized by standardized tests [16].

### Methicillin susceptibility tests and SCCmec typing

Methicillin susceptibility was determined by disk diffusion using the 30 µg cefoxitin disk (CECON; São Paulo, SP, Brazil) according to Clinical Laboratory Standard Institute recommendation (CLSI, 2014). The *Staphylococcus aureus* ATCC 25923 was used as control. Bacterial DNA was extracted by the method described earlier using guanine isothiocyanate [17] and all MRSA isolates were subjected to SCC*mec* typing, according to the method described by Kondo et al. [18].

### PCR for virulence genes

PCR assay targeted 26 virulence genes was performed for all the *S. aureus* isolates, as follows: cytolysins (*hla, hlg, pvl*), SAgs (*sea, seb, sec, sed, see, seg, seh, sei, sem, sen, seo, seu, tst*), exfoliative toxins (*eta, etb*), adhesins (*bbp, cna, ebpS, fnbA, fnbB, eap*) and genes related to biofilm production (*icaA* and *sasG*). The PCR references and control strains are summarized in Table S1 (supplementary material). The primers HLA-1 (5ʹ-AATCCTGTCGCTAATGCC) and HLA-2 (5ʹ-CAGCAATGGTACCTTTCG) used in the PCR for the *hla* gene were designed previously by our group [19] and generated a fragment of 208 pb. Amplification conditions were: denaturation at 94°C for 2 min, followed by 30 cycles of 94°C for 1 min, 55°C for 1 min and 72°C for 1 min, with a final extension at 72°C for 5 min. The expression of the *hld* gene was assessed by measuring the hemolysin activity in blood agar, as described by Harigaya et al. [20]. The RN4220 strain was used as control.

### Biofilm formation

The biofilm formation was evaluated for all isolates on 96-well microtiter polystyrene plates TPP 92,096 (Techno Plastic Products; Trasadingen, Switzerland) as described by Ferreira et al. [21]. Twenty microliters of a bacterial suspension in sterile distilled water corresponding to the 0.5 McFarland standard were added in triplicate to the wells containing 180 µl of tryptic soy broth, TSB (Becton, Dickinson and Company; Sparks, MD, USA), supplemented with 1% glucose (Isofar; Duque de Caxias, RJ, Brazil). The plates were incubated at 37°C for 24 h without shaking. The biofilm was heat-fixed at 60°C for 1 h, stained with 0.1% (w/v) safranin for 15 min, and distained with 95% (v/v) ethanol for 30 min. The biofilm phenotype was categorized as absent, weak, moderate, or strong according to Stepanović et al [22]. Besides that, 13 isolates were randomly chosen in order to investigate biofilm production in wells coated with 50 µg/ml of human plasmatic fibrinogen (Sigma Chemical Company; St. Louis, MO, USA) [23]. All tests were performed in triplicate. *Staphylococcus aureus* ATCC 33591 was used as a control for biofilm formation.

### Genotyping tests

Pulsed-field gel electrophoresis (PFGE) was carried out for all *S. aureus* isolates, after digesting genomic DNA with *Sma*I (New England Biolabs, Inc.; Beverly, MA, USA) by the method described previously [24] with pulse times increasing from 1 to 35 seconds and a running time of 23 hours. The Dice index and the un-weighted pair group method with arithmetic average (UPGMA) with 0.5% optimization and 1% position tolerance were used for similarity and cluster analysis. After PFGE, isolates were grouped in genotypes according to their similarities in band patterns [25] and the clonality was obtained by comparisons with previously published pictures [26]. Multilocus sequence typing (MLST) [27] was performed for one representative isolate of each genotype to determine the sequence type (ST). Internal fragments of seven housekeeping genes have been amplified (*arcC*, encoding carbamate kinase; *aroE*, shikimate dehydrogenase; *glpF*, glycerol kinase; *gmk*, guanylate kinase; *pta*, phosphate acetyltransferase; *tpi*, triosephosphate isomerase; and *yqiL*, acetyl coenzyme A). The allele sequences were analyzed using Bioedit 7.0 software and the MLST database (www.pubmlst.gov). In this technique, each allelle sequence is assigned with a number. The sequence type (ST) (or allelic profile) is based on the combination of the seven assigned numbers [28]. Minimum spanning trees (MST) were created by goeBURST implemented in PHYLOViZ [29] 2.0 software. The STs were represented by circles; the size of a circle is proportional to the number of isolates of this particular ST.

### Statistical analysis

Virulence genes and clonality data were analyzed using SPSS (IBM; Armonk, NY, USA) 21.0 software program for Windows. The Exact Fisher and Chi-Square tests were used to analyze the data. The biofilm data were analyzed using the GraphPad Prism 6.01 program (San Diego, California, USA). The two-way Anova test followed by the Sidak test for multiple comparisons was used in order to evaluate the biofilm formation in the presence of fibrinogen. Significance was established at 5% (*p* < 0.05). Unadjusted associations between independent variables were assessed through odds ratio (OR) and 95% confidence interval (CI).

## Results

### Clinical data

During the period of this study, 106 pediatric patients with AD were attended in IPPMG ambulatory. Among these, 55 (51.9%) presented cutaneous infection caused by *S. aureus*. The clinical data were assessed for 49 patients and revealed that 67% were female. The median age of subjects was 7 years and 29 of the patients (59.2%) presented moderate AD, 11 (22.4%) had mild AD and 9 (18.4%) presented the severe form of the disease. The SCORAD data for 6 (10.9%) patients was not available.

### Methicillin resistance and genotypes

Among the 55 *S. aureus* isolates, 23 (41.8%) were MRSA and 32 (58.2%) were methicillin-susceptible *S. aureus* (MSSA) (Table 1). The clonal complexes found for these 55 *S. aureus* isolates were: 30 (19 isolates; 34.5%), 5 (15 isolates; 27.3%), 1 (12 isolates; 21.8%), 398 (3 isolates; 5.6%); 8 (2 isolates; 3.6%); 15 (2 isolates; 3.6%); 45 (1 isolate; 1.8%) and 97 (1 isolate; 1.8%). The SCC*mec* IV was found in all the MRSA isolates and the PFGE technique helped to cluster the endemic clonal profiles initially. Among the 23 MRSA isolates, 22 (95.6%) were included in three CCs: 30 (USA1100/ST30; 52.2%), 5 (USA800/ST5 and ST83; 30.4%) and 1 (USA400/ST1 and ST188; 13%) (Table 2). One isolate was not related to the MRSA endemic clones and the MLST method showed the CC97. MRSA isolates were found in 45.5%, 41.4% and 33.3% of patients with mild, moderate, and severe AD, respectively, and the occurrence of virulence genes and genetic lineages was balanced among isolates recovered from these patients.

**Table 1.** Association between methicillin resistance, virulence genes, and clonal complex in *Staphylococcus aureus* isolates from infected skin lesions of atopic dermatitis children

*S. aureus* associated aspects	Methicillin-susceptibility nº (%) of isolates			*p* value	Clonal complex^a^ nº (%) of isolates		
	Total (n = 55)	MRSA (n = 23)	MSSA (n = 32)		1 (n = 12)	5 (n = 15)	30 (n = 19)
Virulence Genes							
*pvl*	39 (70.9)	18 (78.2)	21 (65.6)	0.37	8 (66.7)	8 (53.3)	17 (89.4)*
*hld*	29 (52.7)	8 (34.7)	21 (65.6)	0.03*	7 (58.3)	12 (80)	5 (26.3)
*hlg*	54 (98.2)	23 (100)	31 (96.9)	1.00	12 (100)	15 (100)	19 (100)
*eta*	6 (10.9)	3 (13)	3 (9.4)	0.68	0 (0)	2 (13.3)	2 (10.5)
*etb*	4 (7.3)	2 (8.7)	2 (6.2)	1.00	0 (0)	3 (20)	1 (5.2)
*sea*	42 (76.4)	17 (73.9)	25 (78.1)	0.75	11 (91.7)	10 (66.7)	16 (84.2)
*seb*	17 (30.9)	7 (30.4)	10 (31.2)	1.00	7 (58.3)*	1 (6.7)	3 (15.8)
*sed*	1 (1.8)	0	1 (3.1)	1.00	1 (8.3)	0	0
*seg*	28 (50.9)	18 (78.2)	10 (31.2)	0.0009*	4 (33.3)	8 (53.3)	13 (68.4)
*seh*	15 (27.3)	7 (30.4)	8 (25)	0.76	6 (50)*	2 (13.3)	4 (30.8)
*sei*	24 (43.6)	18 (78,2)	6 (18.7)	0.0009*	2 (16.6)	7 (46.7)	14 (73.7)*
*sem*	35 (63.6)	21 (91.3)	14 (43.7)	0.0005*	4 (33.3)	11 (73.3)	16 (84.2)
*sen*	30 (54.5)	19 (82.6)	11 (34.4)	0.0008*	3 (25)	9 (60)	16 (84.2)*
*seo*	27 (49.1)	20 (86.9)	7 (21.8)	0.0001*	1 (8.3)	9 (60)	15 (78.9)*
*tst*	1 (1.8)	0	1 (3.1)	1.00	0	0	0
*bbp*	24 (43.6)	16 (69.5)	8 (25)	0.0021*	2 (16.6)	6 (40)	14 (73.7)*
*cna*	39 (70.9)	14 (60.9)	25 (78.1)	0.23	8 (66.6)	8 (53.3)	16 (84.2)
*eap*	54 (98.2)	23 (100)	31 (96.9)	1.00	11 (91.6)	15 (100)	19 (100)
*ebpS*	51 (92.7)	23 (100)	28 (87.5)	0.13	12 (100)	15 (100)	17 (89.4)
*fnbB*	23 (41.8)	7 (30.4)	16 (50)	0.17	3 (25)	6 (40)	7 (36.8)
Clonal complex							
30	19 (34.5)	12 (52.2)	7 ()(21.8)	0.024*	na	na	na
5	15 (27.3)	7 (30.4)	8 (25)	0.76	na	na	na
1	12 (21.8)	3 (13)	9 (28.1)	0.32	na	na	na

ST – sequence type; ^a^Not included CCs 8, 15, 45, 97 and 398 that accounted for 11 isolates; ^b^Among 49 patients who had clinical data available; na – not applicable; *Statistically significant difference (*p* value <0.05). All isolates were positive to *fnbA, icaA, hla, sasG* and *seu* genes. No isolate was positive to *sec* or *see* genes.

**Table 2.** Characteristics of MRSA isolates recovered from infected skin lesions of atopic dermatitis children

Isolate	Isolation date (yy/mm)	SCORAD/ Severity	Biofilm production	Virulence genes	Clonality/ST/CC
6da	11/09	44/MO	NP	*sea, seg, seh, sei, sem, sen, seo, cna*	USA400/1/1
9da	11/09	20.9/MI	NP	*pvl, sea, seb, seg, sei, sem, sen, seo, bbp, cna*	USA1100/30/30
10da	11/10	38/MO	NP	*pvl, cna*	ND/188/1
14 da	11/10	17.8/MI	WE	*pvl, sea, seg, sei, sem, sen, seo, bbp*	ND/83/5
21 da	11/10	29/MO	NP	*pvl, sea, seg, seh, sei, sem, sen, seo, hld, bbp, cna*	USA1100/30/30
74 da	12/02	24.5/MO	WE	*pvl, sea, seg, sei, sem, sen, seo, hld, bbp, cna*	USA1100/30/30
115 da	12/02	21.5/MI	NP	*pvl, hld, sea, seg, sei, sem, sen, seo, bbp*	USA800/5/5
110 da	12/03	ND	WE	*pvl, hld, sea, seb, sem, bbp*	ND/188/1
111 da	12/03	39.3/MO	NP	*pvl, sea, seg, sei, sem, sen, seo, bbp*	USA1100/30/30
116 da	12/03	26.5/MO	WE	*pvl, sea, seg, seh, sei, sen, seo, bbp, cna, fnbB*	USA1100/30/30
166 da	12/04	14.1/MI	NP	*pvl, sea, seg, sei, sem, sen, seo, bbp, cna, fnbB*	USA1100/30/30
185 da	12/05	18.3/MI	MO	*hld, eta, etb, seg, sei, sem, sen, seo, cna, fnbB*	USA800/5/5
203 da	12/05	ND	NP	*pvl, hld, sea, seg, sei, sem, sen, seo, cna*	USA800/5/5
206 da	12/05	ND	SG	*pvl, seg, sei, sem, sen, seo, bbp, cna*	USA1100/30/30
237 da	12/05	34.7/MO	NP	*seg, sei, sem, sen, seo, bbp, cna*	USA1100/30/30
140 da	12/06	26.7/MO	NP	*pvl, hld, sea, seh, sem, sen, seo, bbp, fnbB*	USA800/5/5
224 da	12/06	44.5/MO	NP	*pvl, sea, seg, sei, sem, sen, seo, bbp, cna, fnbB*	USA1100/30/30
241 da	12/06	62.5/SE	NP	*seg, sei, sem, sen, seo*	USA800/5/5
255 da	12/07	47/MO	NP	*pvl,hld, etb,seb, seg, sei, sem, seo, cna*	USA800/5/5
276 da	12/07	66/SE	NP	*pvl, sea, seg, seh, sei, sem, sen, seo, bbp, fnbB*	USA1100/30/30
286 da	12/07	27/MO	NP	*pvl, sea, seh, bbp, cna, fnbB*	ND/97/97
305 da	12/08	49.5/MO	NP	*sea, seh, sem, sen, seo*	USA1100/30/30
339 da	12/08	64/SE	NP	*pvl, sea, seg, sei, sem, sen, seo, bbp, cna*	USA1100/30/30

MO – moderate; MI – mild; SE – severe; ND – not determined; ST – sequence type; NP – non-producer; WE – Weak producer; SG – Strong producer. *All MRSA isolates presented MIC to trimethoprim/sulfamethoxazole <0.125/2.375, harbored the SCC*mec* IV, were positive to *hla, hlg, seu, fnbA, icaA, sasG* and *eap* genes and were negative to *sec, sed, see* and *tst* genes.

### Virulence genes

**Figure 1.**
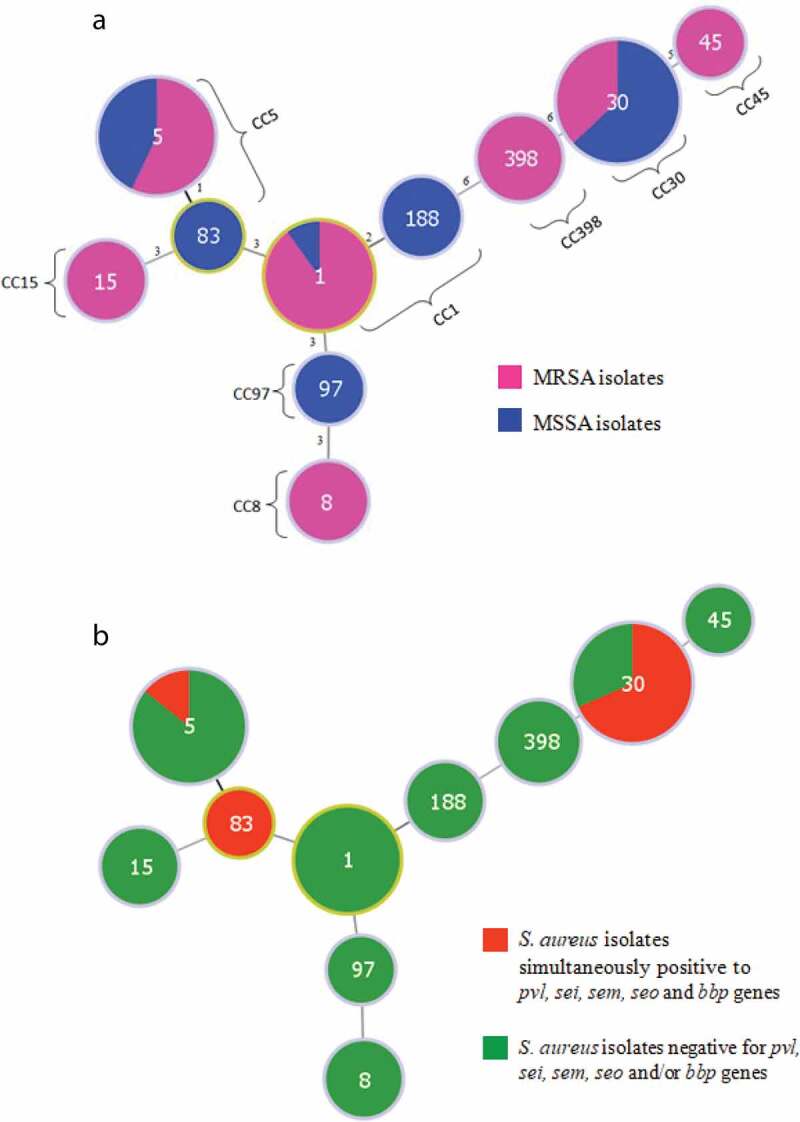
Minimum spanning tree (MST) showing the relationship between different *Staphylococcus aureus* STs assigned by the analysis of MLST data. Each node represents one sequence type, and the corresponding ST is given inside the node. The size of each node is directly proportional to the number of isolates included in that ST. Nodes with highlighted yellow margin show the STs 1 and 83 that are founder members of clonal complexes. (a). MRSA and MSSA isolates belonged to each ST; number of different alleles is labeled between the nodes; (b). *S. aureus* isolates simultaneously positive to *pvl, sei, sem, seo* and *bbp* genes and isolates negative for one or more of these genes. MSSA: methicillin susceptible *S. aureus*; MRSA: Methicillin resistant *S. aureus*; CC: clonal complex

All *S. aureus* isolates were positive to at least one SAg gene among the 26 virulence genes investigated. The *hla, seu, fnbA, icaA* and *sasG* genes were found in all isolates. Besides these genes, the most prevalent rates were found for *hlg* (98.2%) and for the adhesins: *eap* (98.2%), *ebpS* (92.7%), and *cna* (70.9%). High rates were also found for *sea* (73.4%) and *pvl* (70.9%) and for the *egc* cluster-encoded enterotoxins: *sem* (63.6%), *seg* (50.9%), *sen* (54.5%), *sei* (43.6%), *and seo* (49.1%). The *sec* and *see* genes were not found in any isolate, while *tst* and *sed* (1.8%), *etb* (7.3%), and *eta* (10.9%) were found with low frequency. The virulence genes *seo* (*p* = 0.0001), *sem* (*p* = 0.0005), *sen* (*p* = 0.0008), *seg* and *sei* (*p* = 0.0009), *and bbp* (*p* = 0.0021) were significantly more present in the MRSA than in the MSSA isolates. On the other hand, the expression of the *hld* gene was more prevalent between MSSA isolates (*p* = 0.03). The *pvl, sei, sen, seo* and *bbp* genes were mainly found among the CC30 isolates (Figure 1b), while *seb* and *seh* were more frequent within CC1 isolates. The CC30 lineage was significantly more related to MRSA (*p* = 0.024) (Figure 1a).

### Biofilm production

**Figure 2.**
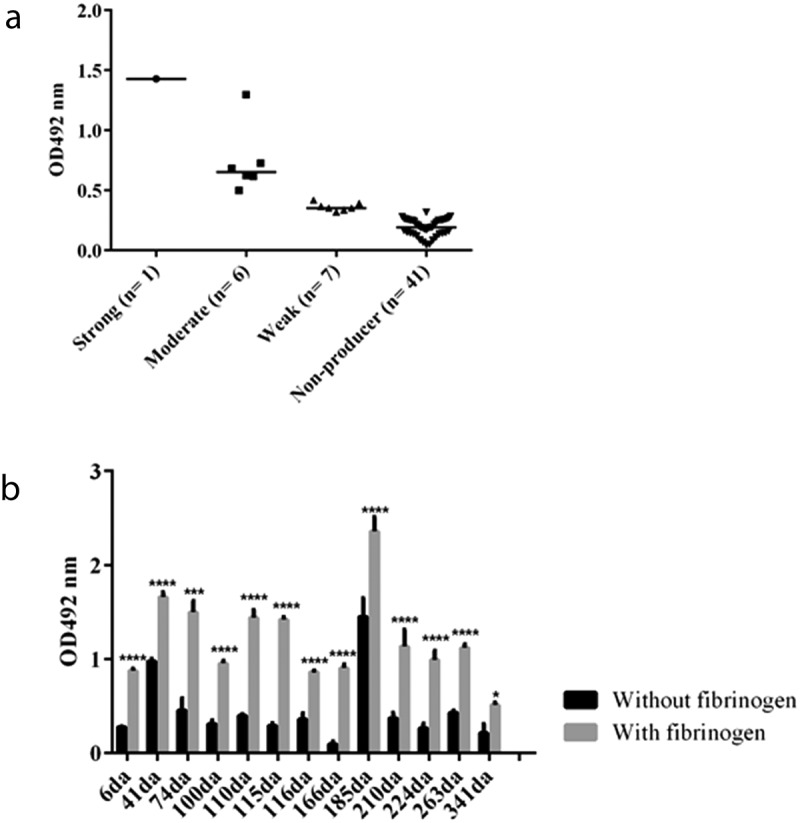
Biofilm production by *Staphylococcus aureus* isolates recovered from infected skin lesions of AD children (a). Distribution of the 55 isolates according to biofilm formation (b). Biofilm formation by 13 representative isolates in the absence and in the presence of human plasmatic fibrinogen

The most part of the 55 *S. aureus* isolates investigated for biofilm formation were categorized as non-producers (41; 74.5%), while 7 (12.7%) were weak producers and 6 (10.9%) moderate producers. Only one isolate was considered a strong biofilm producer (Figure 2a). Interestingly, all the 13 isolates randomly selected with variable profiles of biofilm production presented a significant increase in biofilm formation or only started to produce biofilm when evaluated in wells coated with fibrinogen (Figure 2b).

Non- or weak producers in the absence of fibrinogen: 6 da, 74 da, 100 da, 110 da, 115 da, 116 da, 166 da, 210 da, 224 da, 263 da, 341 da; Moderate producers in the absence of fibrinogen: 41 da and 185 da; OD492 nm – Optical density at 492 nm. The horizontal bars in **A** represent the mean of the values. The black vertical bars in **B** represent the standard deviation values. The symbols *, *** and **** represent, respectively, adjusted *p* values < 0.05, 0.001 and 0.0001.

## Discussion

*Staphylococcus aureus* remains a major concern for AD pediatric patients. The virulence factors produced by this pathogen, such as adhesins, superantigens (SAgs), and cytotoxins seem to play significant roles in the development and behavior of the disease. Here we investigated the features related to *S. aureus* isolates from infected skin lesions in Brazilian children with AD, as well as the relationship of these characteristics with the severity of the disease. Although we did not find any significant association between the severity of the disease and presence of virulence genes, methicillin resistance, or clonality, we detected a high frequency of USA1100/ST30/CC30 MRSA isolates carrying a great number of virulence genes, with a significant number of the *pvl, bbp* and *egc* cluster genes. These epidemiological aspects have profound relevance and can determine the worsening of the disease and the treatment refractoriness.

In this study, a high rate of methicillin-resistance (41.8%) among the *S. aureus* isolates from infected lesions of AD patients was found, whereas in USA, Matiz et al. [30] found only 14% of MRSA isolates in this lesion type. When we compare the colonization rates by MRSA between North American children, which reaches up to 25% [31] and the Brazilian children that is about 45% [32] we can suppose that this difference must be due to the geographic characteristics of each country. Regarding the SCC*mec* type, some authors have showed the predominance of the type IV in MRSA isolates from AD children [5,33]. Interestingly, the SCC*mec* IV, which was found in all isolates in our study, is prevalent in community MRSA isolates recovered in Brazil [34], showing that *S. aureus* isolated from AD patients probably follows the same pattern of isolates recovered from the community in each country.

Molecular analysis of all *S. aureus* isolated in the present study showed that 83.6% of them and 95.6% of MRSA isolates belonged to CCs 1, 5, and 30. This predominance was not a surprise, since these are the major lineages of *S. aureus* found in Brazilian hospitals [35] and community [34]. We also observed the sporadic STs 8, 15, 45, 97, and 398. These findings suggest that isolates from infected lesions may have different origins, not only from widespread lineages but also from the patient’s own microbiota or family [36].

The CC30 was notably the most common lineage found in the present study. However, several studies involving *S. aureus* isolates from the skin of AD patients have shown that CC30 isolates are rare [13,37–39]. An Irish study from Fleury et al. [38] showed that CC1 was the predominant lineage of *S. aureus* on the skin of AD children, while CC30 was prevalent in nasal swabs of healthy children. Geoghegan et al. [40] in a study conducted in Ireland hypothesized that an enhanced adhesion to corneocytes could contribute to the success of the CC1 strain, as well as the possibility of CC1 growing better than CC30 strains in the skin environment. In Spain, when Rojo et al. [13] compared *S. aureus* isolates from AD patients with controls, they found the CC5 lineage as the most prevalent in AD patients, whereas CC30 was the most common lineage among control individuals. Moreover, the analysis of virulence genes showed that isolates from AD patients were notably more virulent when compared to controls. The authors then suggested that the virulence profile could be closely related to the success of a particular lineage in colonize and trigger the infection on atopic skin [13].

Possibly the high frequency of CC30 isolates in our study is related to a great number of virulence factors carried by isolates that belong to this lineage circulating in Brazil. Here, we demonstrate that *pvl, sei, sen, seo,* and *bbp* genes were significantly more frequent in CC30 than in CC1 and CC5. The remarkable association between methicillin resistance and high virulence among CC30 isolates in Brazil may be the key to understand a molecular epidemiology of *S. aureus* recovered from AD in the country. Indeed, CC30 *S. aureus* isolates from South America, including Brazil [41] and Argentina [42] seem to present more virulence features and resistance to methicillin than isolates from that lineage found in AD patients from Ireland [43] and Spain [44], which would confirm our findings.

All 55 *S. aureus* isolates of the present study showed multiple virulence genes. The *hla, seu, fnbA, icaA,* and *sasG* genes were found in all isolates, *hlg* were found in 98.2%, and *cna* in 70.9% of isolates. Aggarwal et al. [45] found similar rates of *hla*¸ *hlg, icaA,* and *cna* in a study with MSSA and MRSA isolated from a variety of infections in India. However, these authors detected different rates of *sea, sec,* and *sei*, which may indicate that some genes are common in *S. aureus*, regardless of the location of the infections, while others may be more or less associated with infections in atopic skin.

Genes of the *egc* cluster ranged from 43% (*sei*) to 63.6% (*sem*) and almost 70% of the isolates presented at least one of these genes. High rates ranging from 65.6% to 71.3% of *S. aureus* isolates carrying at least one of these genes have been associated with non-infected AD lesions [11–13]. In 2004, Holtfreter et al. [46] showed that antibodies for *egc*-encoding enterotoxins inhibited these toxins with a 10- to 100-fold-reduced potency compared with antibodies specific to the classical enterotoxins, indicating a low efficiency in neutralizing *egc*-encoded superantigens by serum factors. The high rates of these genes found in the present study, and the immunological aspects associated to the enterotoxins *egc* cluster may indicate a relevant contribution in AD pathogenesis. In the present study, *egc* cluster and *bbp* genes were more presented among MRSA isolates than in the MSSA. Although MSSA isolates are usually considered to harbor more virulence genes [47], some authors have already shown that *seg* and *sei* genes are significantly more prevalent in MRSA [48]. These findings support the hypothesis that MRSA lineages are evolving over time, acquiring new features, and gaining the ability to trigger infection [49].

The *pvl* gene was detected in 70.9% of the isolates, a percentage much higher than those observed by other authors in studies involving AD in children. Chiu and colleagues [6] in Singapore and Pascolini and colleagues [50] in Italy evaluated *S. aureus* isolates from non-infected AD lesions and found 3% and 2.2% of *pvl* – positive isolates, respectively. Interestingly, among isolates recovered from skin infections in non-atopic patients, the rates of *pvl* genes can reach 90% [51]. This evidence suggests that the PVL plays a relevant role in cutaneous infections, regardless the patient’s health status. However, it is worth to mention that AD patients have chronic wounds that persist for years, making these individuals that are colonized with these strains constantly susceptible to invasive infections.

We did not find any association between a virulence gene or a specific lineage with the severity of AD. Some studies have shown that *S. aureus* isolates that carry any SAg genes are associated with greater severity of AD [10,52]. However, Sag production was not found exclusively in isolates from patients with AD but also among isolates from other patients, suggesting that other virulence factors may be contributing to the severity of AD [52]. It is possible that AD can be aggravated by the action of one of the toxins or by the potentiation of the action of two or more toxins or even by the presence of other aspects associated with the pathology of AD.

This study observed that 13 isolates that produced little or no biofilm presented a significant increase or only started to produce higher amounts of biofilm when they were grown in wells coated with fibrinogen. The role of the biofilm by *S. aureus* in AD is still poorly investigated [14,53,54]. Di Domenico et al. [53] showed that *S. aureus* isolated from skin lesions of AD patients were able to produce high amounts of biofilm, especially the ones isolated from patients with severe AD. Ramundo et al. [54] showed that USA1100/ST30 Brazilian isolates, a prevalent lineage found in the present study, were often considered as weak biofilm producers. In our study, most of the isolates did not produce biofilm even though they presented genes related to its formation. However, in the presence of fibrinogen, a protein that is found in the skin lesions of AD patients due to the scratching caused by constant itching, the biofilm formation occurred. In our study, biofilm was significantly produced by all isolates tested, demonstrating that *in vivo* this virulence factor could favor the colonization and permanence of this species in the skin.

Although all patients enrolled in the study attended the AD outpatient clinic, only 55 children presented skin infections, and most of them presented moderate AD. The low number of subjects and the unbalanced samples of disease severity can affect the statistical analyses. Although there are differences in microbial community between different parts of the body lesions locations were not evaluate. For example, *Staphylococcus* is the most common genus in some moist areas, such as plantar heel, popliteal fossae, and occiput region in AD patients [55, 55–67].

In conclusion, this study showed a high frequency of CC30 MRSA isolates carrying significantly more virulence genes than other lineages, including *pvl* and *egc* cluster genes. Moreover, the isolates considered to be non-biofilm producers started to produce this virulence factor in the presence of fibrinogen. Therefore, this study highlights the epidemiological relevance of the data and its influence on the worsening of the disease and on the impairment of the treatment of AD.

## Supplementary Material

Supplemental Material
