# Supplementary material for: Methicillin-resistant Staphylococcus aureus from infected skin lesions present several virulence genes and are associated with the CC30 in Brazilian children with atopic dermatitis
Source: Virulence. 2021 Jan 7;12(1):260–9. doi: 10.1080/21505594.2020.1869484 (PMC7808431; doi:10.1080/21505594.2020.1869484)
Supplement: Supplemental Material [file KVIR_A_1869484_SM9234.docx]

Table S1 - Virulence genes, oligonucleotides and PCR conditions used in this study to investigate 26 virulence genes in *Staphylococcus aureus* isolated from infected skin lesions of atopic dermatitis children.

| **Gene** | **Primers – sequence (5’-3’)** | **Concentration (µM)** | **Cycle** | **Amplicon (bp)** | **Reference** | **Control strain (Reference)** |
| --- | --- | --- | --- | --- | --- | --- |
| ***pvl*** | PVL1 – ATCATTAGGTAAAATGTCTGGACATGATCCA  PVL2 – GCATCAASTGTATTGGATAGCAAAAGC | 1 | 1 | 433 | Lina *et al*.^51^ | 601a ^56^ |
| ***hlg*** | HLG1 – GCC AATCCGTTATTAGAAAATGC  HLG2 – CCATAGACGTAGCAACGGAT | 1 | 1 | 937 | Lina *et al*.^51^ | 184a  (This study)^*^ |
| ***hla*** | HLA1 – AATCCTGTCGCTAATGCC  HLA2 – CAGCAATGGTACCTTTCG | 1 | 1 | 208 | Caboclo^19^ | 184a  (This study)^*^ |
| ***eta*** | ETA1 – ACTGTAGGAGCTAGTGCATTTGT  ETA2 – TGGATACTTTTGTCTATCTTTTTCATCAAC | 1  0.4 | 11 | 190 | Jarraud *et al*.^57^ | 184a  (This study)^*^ |
| ***etb*** | ETB1 – ACAAGCAAAAGAATACAGCG  ETB2 – GTTTTTGGCTGCTTCTCTTG | 1.2 | 3 | 226 | Jackson *et al.*^58^ | 184a  (This study)^*^ |
| ***sea*** | SEA1 – TTGGAAACGGTTAAAACGAA  SEA2 – GAACCTTCCCATCAAAAACA | 1  0.4 | 5 | 120 | Johnson *et al*.^59^ | 184a  (This study)^*^ |
| ***seb*** | SEB1 – ACATGTAATTTTGATATTCGCACTG  SEB2 – TGCAGGCATCATGTCATACCA | 1.2 | 6 | 667 | LØvseth *et al*.^60^ | ATCC14448 |
| ***sec*** | SEC1 – CTTGTATGTATGGAGGAATAACAA  SEC2 – TGCAGGCATCATATCATACCA | 0.8 | 1 | 283 | Monday *et al*.^61^ | ATCC19095 |
| ***sed*** | SED1 – GTGGTGAAATAGATAGGACTGC  SED2 – ATATGAAGGTGCTCTGTGG | 0.8 | 1 | 384 | Monday *et al*.^61^ | ATCC23235 |
| ***see*** | SEE1 – TACCAATTAACTTGTGGATAGAC  SEE2 – CTCTTTGCACCTTACCGC | 0.8 | 1 | 170 | Monday *et al*.^61^ | ATCC27664 |
| ***seg*** | SEG1 – AATTATGTGAATGCTCAACCCGATC  SEG2 – AAACTTATATGGAACAAAAGGTACTAGTTC | 0.2  1 | 7 | 642 | Jarraud *et al*.^57^ | 184a  (This study)^*^ |
| ***seh*** | SEH1 – CAACTGCTGATTTAGCTCAG  SEH2 – GTCGAATGAGTAATCTCTAGG | 2.4  1.2 | 8 | 360 | Monday *et al*.^61^ | 633a  (This study)^*^ |
| ***sei*** | SEI1 – CTCAAGGTGATATTGGTGTAGG  SEI2 – AAAAAACTTACAGGCAGTCCATCTC | 1  0.2 | 3 | 576 | Jarraud *et al*.^57^ | 184a  (This study)^*^ |
| ***sem*** | SEM1 – CTATTAATCTTTGGGTTAATGGAGAAC  SEM2 – TTCAGTTTCGACAGTTTTGTTGTCAT | 2  0.2 | 9 | 300 | Jarraud *et al*.^57^ | 184a  (This study)^*^ |
| ***sen*** | SEN1 – ATGAGATTGTTCTACATAGCTGCAAT  SEN2 – AACTCTGCTCCCACTGAAC | 2  0.2 | 4 | 680 | Jarraud *et al*.^57^ | 184a  (This study)^*^ |
| ***seo*** | SEO1 – AGTTTGTGTAAGAAGTCAAGTGTAGA  SEO2 – ATCTTTAAATTCAGCAGATATTCCATCTAAC | 2  0.4 | 1 | 180 | Jarraud *et al*.^57^ | 184a  (This study)^*^ |
| ***seu*** | SEU1 – AAACATTAAAGCCCAAGAG  SEU2 – ACACCGCCATACATACAC | 1 | 10 | 243 | Tang *et al*.^62^ | 184a  (This study)^*^ |
| ***tst*** | TST1 – ATGGCAGCATCAGCTTGATA  TST2 – TTTCCAATAACCACCCGTTT | 1 | 1 | 350 | Johnson *et al*.^59^ | 646a  (This study)^*^ |
| ***bbp*** | BBP1 – AACTACATCTAGTACTCAACAACAG  BBP2 – ATGTGCTTGAATAACACCATCATCT | 1.6 | 2 | 575 | Tristan *et al*.^63^ | 1155a ^56^ |
| ***cna*** | CNA1 – GTCAAGCAGTTATTAACACCAGAC  CNA2 – AATCAGTAATTGCACTTTGTCCACTG | 1.6 | 2 | 423 | Tristan *et al*.^63^ | 1117a ^41^ |
| ***eap*** | EAP1 – TACTAACGAAGCATCTGCC  EAP2 – TTAAATCGATATCACTAATACCTC | 1 | 13 | 230 | Hussain *et al*.^64^ | 1155a ^56^ |
| ***ebpS*** | EBPS1 – CATCCAGAACCAATCGAAGAC  EBPS2 – CTTAACAGTTACATCATCATGTTTATCTTT | 1.6 | 1 | 186 | Tristan *et al*.^63^ | 184a  (This study)^*^ |
| ***fnbA*** | FNBA1 – CACAACCAGCAAATATAG  FNBA2 – CTGTGTGGTAATCAATGTC | 1.6 | 12 | 1,362 | Peacock *et al*.^65^ | 1155a ^56^ |
| ***fnbB*** | FNBB1 – GTAACAGCTAATGGTCGAATTGATACT  FNBB2 – CAAGTTCGATAGGAGTACTATGTTC | 1.6 | 2 | 524 | Tristan *et al*.^63^ | 1155a ^56^ |
| ***icaA*** | icaAF – AAACTTGGTGCGGTTACAGG  icaAR– TCTGGGCTTGACGTTG | 0.6 | 14 | 750 | Martin-Lopez *et al.*^66^ | 1117a ^41^ |
| ***sasG*** | sasGF – CGCTGATCAGAGATAAGAAAGGACCGG  sasGR – CGCTGATCATTAATTCTTTCTTCTACGAG | 0.6 | 15 | 311 | Roche *et al.*^67^ | 526a ^41^ |

Cycle condition: **1:** 94°C/1min; 55°C/1min; 72°C/1min – 30x; **2**: 94°C/1min; 55°C/min; 72°C/1min – 35x; **3**: 94°C/1min; 56°C/5s; 72°C/50s – 30x; **4**: 94°C/1min; 56°C/1min; 72°C/1min – 30x; **5**: 94°C/1min; 56°C/40s; 72°C/1min – 30x; **6**: 94°C/1min; 59.5°C/45s; 72°C/1min – 30x; **7**: 94°C/40s; 58°C/50s; 72°C/1min – 30x; **8:** 94°C/1min; 53.5°C/45s; 72°C/1min – 30x; **9**: 94°C/1min; 58.5°C/1min; 72°C/1min – 30x; **10**: 94°C/40s; 52°C/5min; 72°C/50s – 35x; **11**: 94°C/1min; 57 C/1min; 72°C/1min – 30x; **12**: 94°C/1min; 50°C/1min; 72°C/2min – 30x; **13**: 95°C/50s; 50°C/1min; 72°C/1min – 30x; **14:** 95°C/30s; 50°C/90s; 72°C/45s – 35x; **15**: 94°C/1min; 50°C/1min; 72°C/1min – 30x. ^*^Presence of the gene confirmed by PCR and sequencing.
